# Three-dimensional Reconstruction of the Microstructure of Human Acellular Nerve Allograft

**DOI:** 10.1038/srep30694

**Published:** 2016-08-01

**Authors:** Shuang Zhu, Qingtang Zhu, Xiaolin Liu, Weihong Yang, Yutao Jian, Xiang Zhou, Bo He, Liqiang Gu, Liwei Yan, Tao Lin, Jianping Xiang, Jian Qi

**Affiliations:** 1 Department of Microsurgery, Orthopedic Trauma and Hand Surgery, the First Affiliated Hospital of Sun Yat-sen University, Guangzhou 510080, PR China; 2 Center for Peripheral Nerve Tissue-engineering and Technology Research Guangdong, Guangzhou 510080, PR China; 3 DSAPM and PCFM Lab, School of Chemistry and Chemical Engineering, Sun Yat-Sen University, Guangzhou 510275, PR China; 4 Department of Prosthodontics, Guanghua School of Stomatology, Sun Yat-sen University, Guangzhou 510080, PR China

## Abstract

The exact inner 3D microstructure of the human peripheral nerve has been a mystery for decades. Therefore, it has been difficult to solve several problems regarding peripheral nerve injury and repair. We used high-resolution X-ray computed microtomography (microCT) to scan a freeze-dried human acellular nerve allograft (hANA). The microCT images were then used to reconstruct a 3D digital model, which was used to print a 3D resin model of the nerve graft. The 3D digital model of the hANA allowed visualization of all planes. The magnified 3D resin model clearly showed the nerve bundles and basement membrane tubes of the hANA. Scanning electron microscopy (SEM) was used to analyse the microstructure of the hANA. Compared to the SEM images, the microCT image clearly demonstrated the microstructure of the hANA cross section at a resolution of up to 1.2 μm. The 3D digital model of the hANA facilitates a clear and easy understanding of peripheral nerve microstructure. Furthermore, the enlarged 3D resin model duplicates the unique inner structure of each individual hANA. This is a crucial step towards achieving 3D printing of a hANA or nerve that can be used as a nerve graft.

It is well acknowledged that the peripheral nerve autograft is the gold standard for reconstruction of peripheral nerve gaps. However, the disadvantages of this procedure, which include donor-site dysfunction, excess scars, and prolonged operation time^1^,^2^,^3^,^4^, are serious factors that surgeons consider. For years, a variety of FDA approved substitutes for autografts have been explored, such as nerve conduits and human acellular nerve allografts^5^,^6^. However, nerve conduits have drawbacks, such as limited use for segmental nerve defects smaller than 30 mm and unacceptable functional results in the reconstruction of motor nerves and mixed nerves^7^. The human acellular nerve allograft is a safe and effective nerve scaffold because it maintains most of the natural structure and micro-bioenvironment of the human peripheral nerve, with no immunogenicity^8^. Nevertheless, it is associated with many drawbacks, such as limited sources and applicable lengths. Given these challenges, a new nerve repair scaffolding should be developed to address these problems and enhance nerve regeneration. From a structural perspective, the natural extracellular matrix (ECM) of peripheral nerves consists of various interwoven protein fibres with diameters ranging from tens to hundreds of nanometres. The nanoscale structure of the ECM offers a natural network of nanofibres to support cells and present an instructive background to guide cell behaviour. Therefore, it is important to find a scaffold that mimics the micro-structure and micro-environment of peripheral nerves.

In recent years, 3D biofabrication has been applied to soft tissue and organ reconstruction in a variety of ways. Tissue-engineered cardiac tissue and ear^9^ and vascular grafts^10^ are a few examples. However, these tissue-engineered soft tissues are biofabricated almost entirely using a computer-designed model that cannot replicate the micro-structure of the soft tissue. An accurate 3D digital model can be acquired through CT or MRI, but a high-resolution imaging database is exceedingly difficult to obtain.

Much progress has been made in the application of confocal optical microscopy and serial block face scanning electron microscopy to visualize the 3D structures of soft tissues^11^,^12^. However, this allows only a limited volume of biological tissue to be visualized. Magnetic resonance imaging (MRI) is a very sensitive and effective tool for soft tissue imaging, but the resolution of MRI is not sufficient to visualize the microstructure. MicroCT imaging is sensitive enough to create high-resolution images for hard tissue, such as bone. However, soft tissue cannot be viewed clearly on microCT because it has a high water content. To improve the contrast of biological tissue in microCT imaging, a number of contrast agents have been used to label the target tissue and image the microstructure more clearly^13^,^14^,^15^,^16^. Unfortunately, the resulting images and 3D microstructures of these digital models are insufficient to allow 3D printing.

We assume that if we eliminate the water in soft tissue, the dried tissue can be scanned by microCT. Furthermore, we believe that microCT imaging followed by 3D reconstruction and printing of the hANA will allow accurate recreation of the cross sectional area of the human acellular nerve allograft (hANA) along its entire length. Furthermore, this analysis will display improved accuracy and greater detail than SEM. In this study, we used microCT to scan a segment of freeze-dried hANA and then printed a magnified resin model of the hANA according to the imaging data. In addition, the characteristics of the transverse section of the freeze-dried hANA were analysed by SEM.

## Results

### Sample preparation

**Figure 1:**
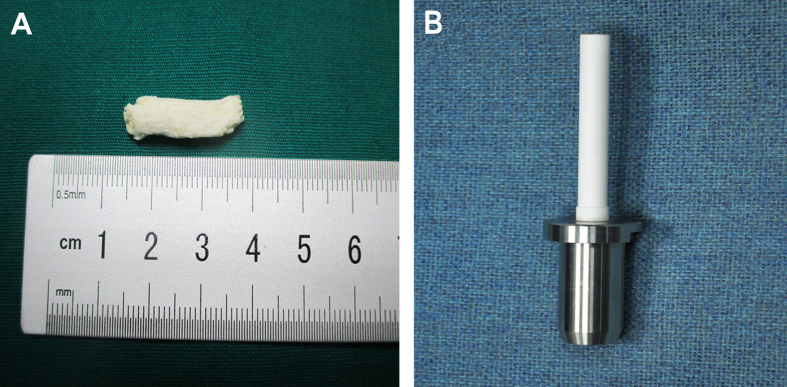
Scanning sample. A segment of human acellular nerve allograft (hANA) was freeze-dried before scanning. The freeze-dried hANA (**A**) should be placed vertically in the white cylinder (**B**). The freeze-dried hANA was solid, it did not shrink when scanned consecutively in the microCT.

As shown in Fig. 1A, a small length of hANA was prepared through lyophilisation, preventing any significant shrinkage. Before scanning, the sample was placed in a small white cylinder (Fig. 1B), and the white cylinder was placed upright during scanning. Because the freeze-dried hANA was solid, it did not shrink in the cylinder as most soft tissue would.

### MicroCT scanning and image analysis

**Figure 2:**
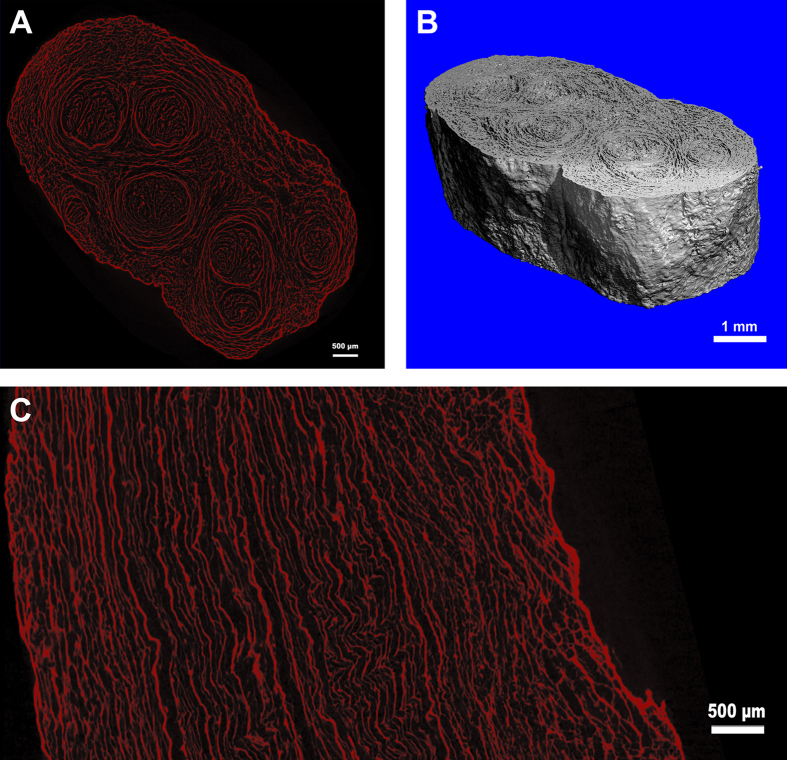
2D and 3D images with a voxel size of 3 μm. (**A**) Transverse images of the hANA, epineurium, perineurium and fasciculus could be clearly identified; (**B**) 3D digital model of the hANA segment. The digital model was reconstructed from 908 horizontal consecutive DICOM images of the hANA, the voxel size of the digital model was 3 μm (i.e., each slice was composed of cubic voxels, the side length of the cubic voxels was 3 μm). The physical size was 7.79 mm × 6.92 mm × 2.72 mm. The reconstructions of the epineurium and fasciculus were clearly seen; (**C**) The longitudinal plane of the hANA. We can distinguish the consecutive fasciculus, perineurium and basilar membrane.

**Figure 3:**
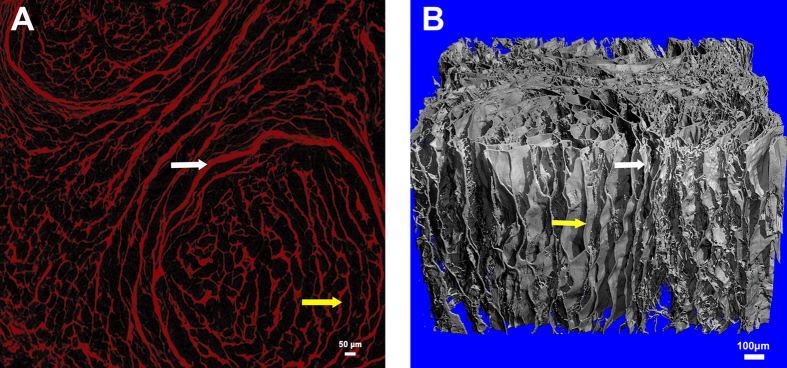
2D and 3D images with a voxel size of 1.2 μm. (**A**) Transverse images of the hANA, perineurium (white arrow) and endoneurial tube (yellow arrow) are displayed; (**B**) 3D digital model of the hANA. The digital model was reconstructed from 908 horizontal consecutive DICOM images of hANA. The voxel size of the digital model was 1.2 μm (i.e., each slice was composed of cubic voxels, the side length of the cubic voxels was 1.2 μm). The physical size was 1.86 mm × 1.44 mm × 1.09 mm. The perineurium (white arrow) and the wall of the endoneurial tube (yellow arrow) were clearly observed.

The sample was scanned in two different voxel sizes: 3 μm and 1.2 μm. In the transverse image with a 3-μm resolution (Fig. 2A), the epineurium, perineurium, fasciculus and endoneurial tube were clearly visible. In total, 908 consecutive 3-μm resolution images were reconstructed, and a 3D digital model of the hANA was obtained (Fig. 2B). The surface of the 3D digital model was smooth, and the epineurium and fasciculus could be clearly observed. One of the distinct advantages of 3D digital model reconstruction was that it allowed investigation of the digital hANA in multiple planes. In the vertical image (Fig. 2C), we could recognize consecutive fasciculi in a longitudinal plane, which was also true for the consecutive perineurium and basilar membranes. In the transverse image with a 1.2-μm resolution (Fig. 3A), the epineurium and endoneurial tube could be clearly identified. The 3D digital model (Fig. 3B) that was reconstructed with 912 slices displayed the consecutive epineurium and the wall of the endoneurial tube.

### 3D printing

**Figure 4:**
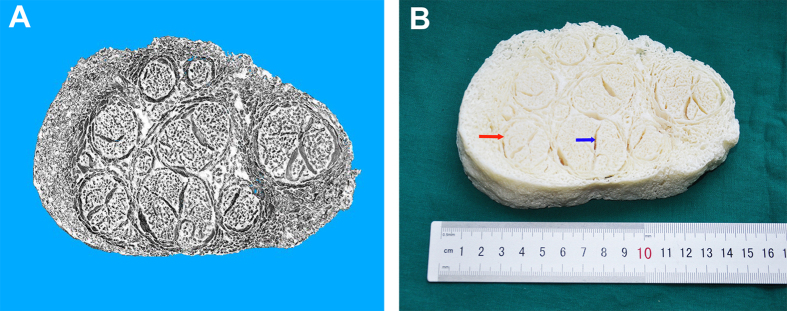
3D printing of hANA. (**A**) STL file of the hANA. This STL file was derived from 200 consecutive slices of the DICOM image with a resolution of 3 μm; (**B**) 3D model of the hANA. This model was fabricated by 3D printing machine using a resin material. The size of the 3D model was 1000 times as large as the hANA, fasciculus (red arrow) and septum (blue arrow) were clearly observed.

A 200-layer STL digital model of the freeze-dried hANA was obtained (Fig. 4A). Because the 3D printer did not reach the resolution of the original digital model, a magnified version was printed based on the STL file at 1000x the size of the digital model. From the physical model, we could easily differentiate the fasciculus, septum and endoneurial tube.

### Scanning electron microscopy

**Figure 5:**
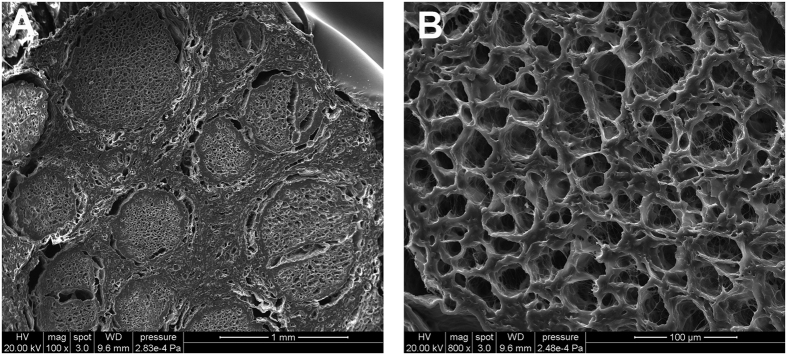
SEM images of freeze-dried hANA. The epineurium, perineurium and fasciculus were clearly visible in the image at a magnification of 100 (**A**); We could distinguish the endoneurial tube and nanofibrous structure in the image at a magnification of 1500 (**B**).

To determine whether the microCT images were compatible with SEM photography, SEM was performed after microCT scanning. The transverse plane of the freeze-dried hANA displayed rich details such as epineurium, perineurium, fasciculus and endoneurial tube at a magnification of 100 (Fig. 5A). When it was changed to a magnification of 1500, the nanofibrous structure of the endoneurial tube could be observed (Fig. 5B).

## Discussion

For decades, people have attempted to reconstruct the substructure of the peripheral nerve. In 1945, Sunderland^17^ first drew 3D graphics of the intraneural structures of peripheral nerves by hand. Even without a computer, the structure of his drawing is very similar to what has been reconstructed by computers today. In 1991, Watchmaker and Gumucio^18^ began using computers to reconstruct nerve bundles; however, they acquired only a rough 3D block picture because the image registration and segmentation were still performed manually. However, the accuracy of this 3D nerve model was inadequate because the running path of the fasciculus could not be demonstrated. Liu *et al*.^19^ and Sun *et al*.^20^ attempted to reconstruct the internal microstructure of the human ulnar and median nerves, respectively. They obtained large numbers of nerve sections that were stained using the AChe histochemical method, and these sections were subsequently scanned. Image post-processing was conducted using computer software. Qi *et al*.^21^ also used a similar method to complete 3D visualization of the common peroneal nerve. Their outstanding work enabled a more accurate and detailed understanding of the anatomy for nerve repair, especially for fascicular nerve repair. However, a few disadvantages still exist regarding this method: i) a large amount of the sectioning, staining and post-processing of the images was very labour-intensive and time-consuming; ii) the reference point of each slice was unstable; therefore, it was difficult to bring all of the images into alignment; and iii) each section was usually sliced with an interval of at least 100 μm. When thousands of images of successive sections were put together in the axial direction using computer software, the nerve bundles or nerve trunk seemed to be discontinuous, which resulted in obvious deviation along the longitudinal axis of the nerve.

MRI and CT were effective tools for obtaining successive images of tissue. Thus, registration and alignment may be possible with computer generated 3D reconstruction of MRI or CT images. MRI was more sensitive for soft tissue imaging than CT. Theoretically, MRI would therefore be more suitable for nerve imaging. Meek *et al*.^22^ developed an *in vivo* three-dimensional visualization of the human median nerve using magnetic resonance diffusion tensor imaging. However, the precise 3D distribution of the fascicles in the median nerve could not be visualized. Richard *et al*.^23^ used diffusion tensor imaging at 4.7 T to visualize acute traumatic peripheral nerve injury. With this technique, only the outlines of injured nerve bundles at the injury site could be recognized; the fasciculus in the nerve trunk could not be distinguished. The spatial resolution of MRI is limited, and an imaging technique with a higher resolution is necessary for 3D visualization of nerve microstructure.

Generally, microCT has been used to view the structures of hard calcified tissues at a cellular to subcellular resolution, i.e., a micrometer to submicrometer resolution. Because X-rays interact with electrons in the sampled object, electron-dense structures can be visualized in an X-ray image. For electron-poor structures, such as soft tissue, contrast agents can be added to increase the contrast against the background because they contain elements with high atomic numbers (high-Z), such as iodine or barium, that absorb X-rays efficiently. This method made 3D reconstruction of soft tissue possible using microCT. Tracy *et al*.^24^ infiltrated the normal sciatic nerve with iodine and obtained an image of the nerve using micro-CT. The nerve tissue showed high contrast, but the microstructure could not be differentiated.

Freeze-drying is a sublimation process that removes moisture from materials at low temperatures while maintaining their structure, bioactivity, and other properties^25^. Based on the sublimation phenomenon, the freeze-drying process should transform the hANA from a soft tissue (electron-poor) into a relatively hard tissue (electron-dense).

In our study, we used the freeze-drying technique to increase the hANA contrast of the microCT image. We found that the lyophilized nerve tissue had an unexpectedly high contrast against air in the absence of contrast agents. When the scanning voxel size was set to 3 μm, we could clearly see the epineurium, perineurium, fasciculus and endoneurial tube in the horizontal plane of the microCT image. This was the first report of a transverse microCT image of a peripheral nerve that displayed as many details as an hematoxylin-eosin staining image. A total of 908 consecutive images with a resolution of 3 μm were reconstructed and then visualized in a 3D visualization system. The reconstructed digital model was 7.79 mm × 6.92 mm × 2.72 mm in size. The surface of the digital model was just as smooth as the real freeze-dried hANA. In the longitudinal section of the 3D model, the continuous nerve bundles were easy to differentiate. When the scanning voxel size was 1.2 μm, the transverse section distinctly displayed the endoneurial tube and allowed precise visualization of the consecutive wall of the endoneurial tube in the vertical plane of the 3D model. From the 3D model, we could determine that the overwhelming majority of the endoneurial tubes were undamaged by the decellularization process.

It is well known that autogenous nerve transplantation is a recommended treatment option for peripheral nerve defect, but donor-site morbidity remains a serious consideration for surgeons and patients. However, a more serious consideration that is often neglected is that the microstructure of the donor nerve does not match that of the recipient nerve. Considering the problems mentioned above, the tissue-engineered nerve graft has some distinct advantages: i) the source of the tissue-engineered nerve graft can be infinite, and ii) the microstructure of the tissue-engineered nerve graft can be created to match that of any injured nerve. The prerequisite of a tissue-engineered nerve graft and its specificity for an injured nerve is that the internal microstructure of the nerve must be acquired. We have successfully reconstructed a 3D digital model of an acellular nerve graft, and by using 3D printing technology, we fabricated an accurate and precise 3D replica of nerve anatomy. Because the exact anatomic details are displayed on a magnified resin model, this makes the tissue-engineered nerve graft one step closer to reality. The next areas that will require greater improvement are printing precision and the printing material.

The freeze-dried hANA was segmented, viewed by SEM and displayed on a 2D plane to determine whether the microstructure was damaged by the freeze-drying process. When viewed at a magnification of 100, the epineurium, perineurium and fasciculus were easily differentiated, and the microCT image also showed the outline of the epineurium, perineurium and fasciculus. To our surprise, the nanofibrous structure of the endoneurium was obvious when viewed at a magnification of 1500, which indicates that the freeze-dried hANA was a highly porous structure. Unlike SEM, the nanofibrous structure could not be imaged with microCT. However, this was of no concern to us because we focused on the 3D reconstruction of the endoneurial tube, and the nanofibrous structure can be developed by the material itself, such as the ECM. In addition, the axial microCT images were better for 3D reconstruction.

The 3D reconstruction and 3D printing of soft tissues and organs are important opportunities and challenges in the field of tissue engineering. In our current research, we confirmed the accurate and precise reproduction of nerve microstructure. MicroCT images displayed explicit details of hANA microstructure along any plane after the 3D digital model was reconstructed and a 3D resin model of the hANA was printed. Finally, the microCT image was consistent with the SEM image. The freeze-drying and microCT scanning techniques are effective for 3D reconstruction of the hANA. In the future, this technique can be applied to reconstruct other soft tissues or organs.

## Materials and Methods

### Preparation of freeze-dried hANA

All experimental protocols were approved by the University of Sun Yat-sen Institutional Review Board. Informed consent was obtained from all subjects. Additionally, the methods were carried out in accordance with the approved guidelines. The human acellular nerve allograft was bought from Guangzhou Zhongda Medical Devices Company (Guangzhou, Guangdong Province, China; Approval No. (2012)3460641). The hANA was frozen at −40 °C for 2 h and transferred to a freeze-drying machine, in which the water inside the frozen hANA was sublimated under a pressure of 0.105 Pa and a temperature of −20 degrees. The freeze-drying process lasted for 5 days.

### MicroCT scanning

The experiment was conducted in a μCT 50 compact cabinet microCT scanner (SCANCO Medical AG, Bassersdorf, Zurich, Switzerland). The sample was scanned in two different settings. The settings for the field of view of 9 mm were as follows: energy/intensity, 55 kVp, 109 μA, 6 W; filter, 0.1 mm Al; calibration, 55 kVp, 0.1 mm Al; BH, Plexi (PMMA); integration time, 1500 ms; average data, 3; FOV/diameter, 10.2 mm; voxel size, 3 μm; Proj/180°, 1500. The settings for the field of view of 4 mm were as follows: energy/intensity, 55 kVp, 72 μA, 4 W; filter, 0.1 mm Al; calibration, 55 kVp, 0.1 mm Al; BH, Plexi (PMMA); integration time, 1500 ms; average data, 3; FOV/diameter, 4.0 mm; voxel size, 1.2 μm; Proj/180°, 1500. After scanning, the two-dimensional images were exported as DICOM files.

### Image analysis

The images of the horizontal cross-sectional layers of the specimen were saved in the DICOM format and imported into MATLAB^®^ version 7.12.0.635 R2011a (MathWorks, Natick, MA, USA) as a data sequence and then viewed in 3D using the orthogonal viewing function. 908 horizontal cross-sectional layers with a resolution of 3 μm were reconstructed for the hANA with a slice increment equal to the pixel size (i.e., each slice was composed of cubic voxels), covering up to 2.72 mm of the length of the sample. Vertical cross-sectional layers were obtained by building up layers of the horizontal cross-sectional layers in MATLAB. 3D images were generated by thresholding the slices using MATLAB.

### 3D printing

Two-hundred DICOM images of freeze-dried hANA with a resolution of 3 μm were imported into MATLAB^®^ version 7.12.0.635R2011a (MathWorks, Natick, MA, USA), and the continuous density was extracted and converted into the standard triangulated language (STL) format using MATLAB software. 3D fabrications were performed using a rapid prototyping apparatus (Objet24 3D Printer Systems, Stratasys, Minneapolis, MN, USA) using acrylic compounds (Object Support SUP705, Stratasys, Minneapolis, MN, USA). The 3D model was printed at a magnification of 1000, and the layer thickness was 28 μm.

### Scanning electron microscopy (SEM)

After microCT scanning, a small section of the freeze-dried hANA was cut into thin slices, and the surface of the slice was sputter-coated with gold. The samples were then imaged using SEM (JSM- 7600F, JEOL, Japan).

## Additional Information

**How to cite this article**: Zhu, S. *et al*. Three-dimensional Reconstruction of the Microstructure of Human Acellular Nerve Allograft. *Sci. Rep.* **6**, 30694; doi: 10.1038/srep30694 (2016).
